# Intrinsic cortical geometry is associated with individual differences in local functional organization

**DOI:** 10.21203/rs.3.rs-9200088/v1

**Published:** 2026-04-08

**Authors:** Francesco Alberti, Pierre-Louis Bazin, R. Austin Benn, Robert Scholz, Wei Wei, Alexander Holmes, Victoria Shevchenko, Ulysse Klatzmann, Carla Pallavicini, Robert Leech, Daniel S. Margulies

**Affiliations:** 1. Université Paris Cité, INCC UMR 8002, CNRS, Paris, France; 2. Centre for Integrative Neuroimaging (OxCIN), FMRIB, Nuffield Department of Clinical Neurosciences, University of Oxford; 3. Full Brain Picture Analytics, Leiden, Netherlands; 4. Wilhelm Wundt Institute for Psychology, Leipzig University, Leipzig, Germany; 5. Max Planck School of Cognition, Leipzig, Germany; 6. National Scientific and Technical Research Council (CONICET), Argentina; 7. Cognitive Neuroscience Center, University of San Andres, Buenos Aires, Argentina; 8. Department of Neuroimaging, King’s College London, UK

## Abstract

It is widely accepted that the geometry of the cerebral cortex constrains its functional topography. However, how geometric properties contribute to individual differences in cortical organization has not been fully characterized. Here, we investigate whether cortical function varies with cortical geometry across individuals at the local or global scale. We characterize functional organization using the first three gradients of functional connectivity, and project individual surfaces into a shared embedding that captures their intrinsic geometry. Fitting localized spatial models within this embedding via lattice Kriging, we test whether interindividual variation of the gradients is linked to differences in spatial location. These models capture a common spatial structure underlying local gradient transitions across individuals, but do not capture differences in global gradient layout. This suggests that universal geometric properties shape the functional transitions between otherwise stable functional systems, meaningfully contributing to subject-specific functional topography.

## Introduction

Spatial proximity is one of the fundamental mechanisms driving functional organization in the cortex: functional differentiation between regions increases with their physical distance^1–3^. Various processing modalities display a clear spatial structure along the cortex, where the spatial arrangement of regions reflects a gradual progression along a processing hierarchy^1,4^. This also applies to global functional organization, with large-scale networks exhibiting a spatial-dependent topography^5,6^. Functional connectivity gradients capture this phenomenon well, showing that the dominant features of cortical organization display gradual transitions between maximally different systems^3^.

The role of spatial proximity emerges from spatially dependent developmental processes—such as chemical gradients and Hebbian learning^1^—and from the propagation of neural activity on the cortical sheet^7^. As a result, both structural and functional connectivity in the adult brain are markedly space-dependent; the physical and topological distance between regions is strictly related.

Based on these premises, the intrinsic functional organization of the cortex should vary with its intrinsic geometry—the distance between regions measured along the surface^8^. Previous studies, for example, show that the varying topography of resting-state networks contributes substantially to how the temporal correlation of their activity changes across individuals^9,10^. Despite not directly assessing intrinsic cortical geometry, these results imply that the relative position of regions on the cortical surface contributes to differences in their intrinsic connectivity profile.

Other evidence, however, shows that individual cortical shape and interregional distances do not explain more variance than group average data when modeling task activity^11,12^. These studies did not directly investigate intrinsic functional organization, yet they highlight how certain geometric features might be too consistent across individuals to determine meaningful functional differences. This might be due to the large spatial scale of task activation maps—at such a scale cortical shapes may be too similar across individuals. Variability of macroscale patterns of functional organization, instead, likely emerges from the interaction of spatial relationships and other features (e.g., structural connectivity)^13,14^.

Thus, despite extensive evidence showing associations between different aspects of cortical geometry and function^3,6,9,11,15–17^, it is still unclear to what extent this association contributes to interindividual variability in functional organization. To expand on this literature, our study models this relationship as a cross-individual association between intrinsic cortical geometry and intrinsic cortical organization. Specifically, we investigate whether geometry relates to how functional differentiation unfolds across the cortex at a local or global scale.

To this end, we first derived representations of functional organization and cortical geometry that are comparable across individuals using structural and functional MRI data. Vertex functional profiles were represented as the first three functional connectivity gradients, which are stable axes of functional organization consistent across subjects and conditions^18^. Cortical geometry, instead, was summarized by the three dominant latent dimensions of the geodesic distance between vertices. These define a shared geometry embedding that captures differences in vertex location across individuals but relative to their intrinsic cortical spaces. As a result, the geometry embedding effectively acts as a common space where we could assess the cross-individual spatial structure of gradients. Specifically, we fitted spatial models of the gradients within searchlights of the geometry embedding after centering them on an individual gradient baseline highlighting local organization. For comparison, we repeated this process on uncentered gradients as well. These two types of models capture the shared spatial structure of local and global trends of functional differentiation.

The results show that individual cortical geometry is systematically related to local functional organization in areas of sharp functional transition. Its association with global functional patterns, instead, only captures group average gradient topography. In other words, geometry mostly relates to the spatial rate at which function changes throughout the cortex, modulating the relative functional similarity between vertices and macroscale functional systems. Modeling this as a general effect across individual surfaces and functional architectures underscores the fundamental nature of geometry’s role in cortical organization.

## Results

**Figure 1 |:**
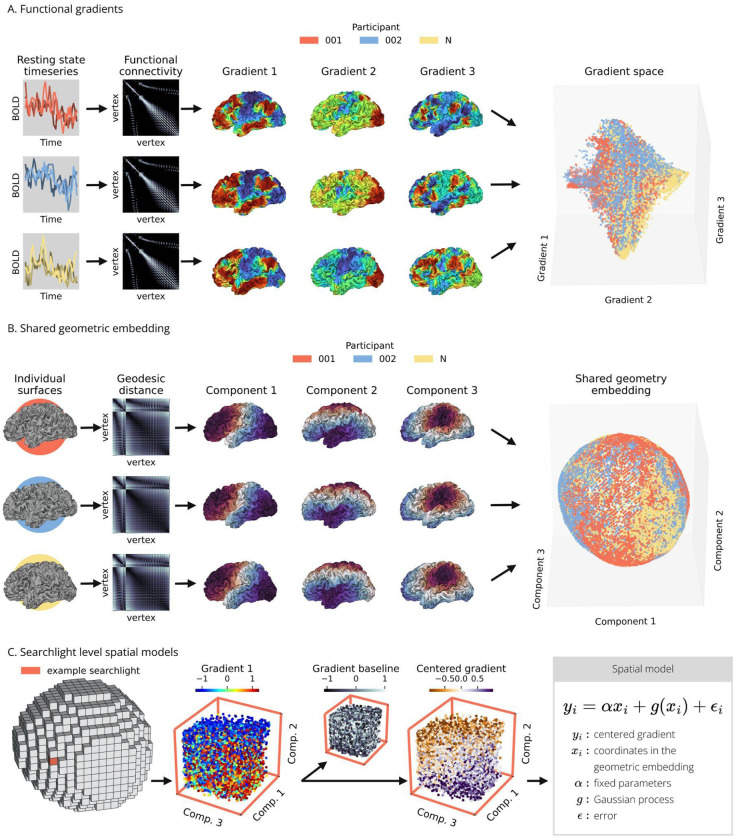
Spatial modeling of functional gradients across individuals **A.** Functional gradients represent dominant axes of cortical organization. From individual resting-state fMRI timeseries we generated vertex-wise functional connectivity matrices, which were in turn decomposed through joint embedding ^19^ to obtain three functional axes, the gradients. **B.** The shared geometry embedding represents vertex placement on individual cortices. From individual cortices, we computed vertex-wise geodesic distance matrices, which we decomposed using joint Laplacian eigenmaps to obtain three components that define a shared representation of intrinsic cortical geometry. **C.** The shared geometry embedding was divided in cubic searchlights. Within each searchlight, the original functional gradients were centered on the subject level mean. Localized spatial regression modeled the association between centered gradients and vertex location across individuals.

Our aim was testing whether cortical geometry is systematically associated with differences in functional organization across individuals. To address this question, we adopted an analytic framework that combines representations of intrinsic cortical geometry and functional organization that are shared and comparable across individuals. Cortical geometry was represented using the leading latent dimensions of vertex-wise geodesic distance, yielding a shared geometry embedding that reflects vertex cortical position across individuals (Fig. 1A). Functional organization was summarized using the first three gradients of functional connectivity^3^, which capture three dominant axes of cortical differentiation: a unimodal-transmodal axis, a visual-somatomotor axis, and task positive-task negative axis (Fig. 1B).

To focus on relative functional differences at a fine spatial scale, we used a searchlight approach. Within each searchlight, we centered gradient values on individual gradient baselines defined as the subject level means, thereby removing large-scale individual offsets and emphasizing local functional organization (Fig. 1C). These centered gradients reflect the arrangement of vertices relative to local functional organization, capturing how functional profiles change in space rather than describe them in absolute terms.

Finally, we modeled geometry-dependent functional variability across individuals using spatial regression in the shared geometry embedding. We fitted a spatial model of the gradients inside each searchlight to estimate localized spatial structures that explain functional variation across individuals (Fig. 1C).

### Geometry explains local gradient variability

**Figure 2 |:**
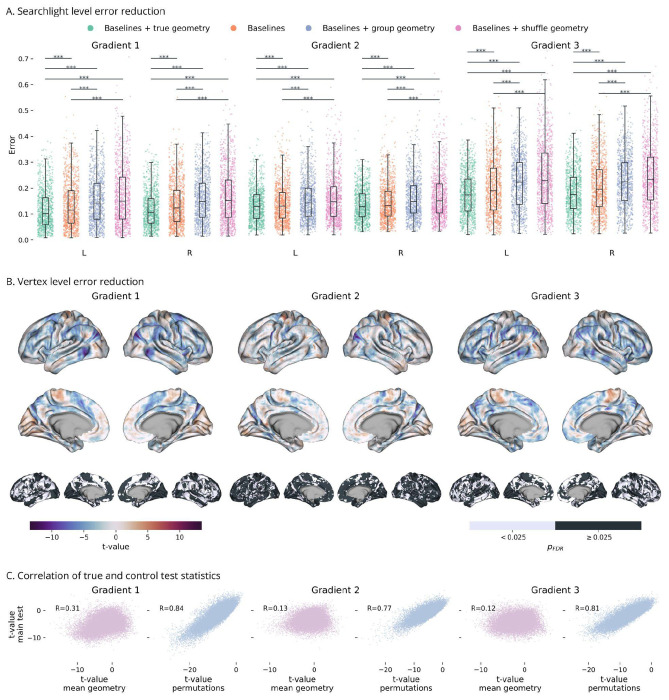
Error-reduction tests **A.** Box plots of searchlight-wise errors (mean absolute error) from the different iterations of the centered models of gradients one to three in the left (L) and right (R) hemisphere. Adding geometry-based predictions to the individual gradient baselines significantly reduces estimate errors, but shuffling geometry embeddings across subjects or replacing the group average has the opposite effect. ***: p<0.001. **B.** T-value maps from the vertex-wise comparison between the error of the baselines alone and combined with the spatial model predictions (top). Negative (blue) values indicate a lower error for the combined estimates. Significance maps in the bottom row. **C.** Scatterplots of t-values from the main vertex-wise tests, and the control tests: true geometry versus group average (pink), true geometry versus cross-subject shuffling (blue). Positive correlations indicate that the contribution of spatial models is strongest where the models are more sensitive to alterations in geometry.

Vertex placement in the shared geometry embedding was found to contribute significantly to interindividual variability of functional gradients. Adding the geometry-based predictions of the centered gradients to the individual gradient baseline significantly reduced searchlight-wise errors compared to the baselines alone (Fig. 2A; Supplementary Table 1). Across all three gradients, the spatial models were able to capture a shared spatial structure of gradient variation that spans across individuals in the geometry embedding.

These whole-cortex tests show that intrinsic cortical geometry meaningfully contributes to gradient variability between individuals, as functional differences are not fully captured by the individual gradient baselines.

By contrast, when gradients were not centered before modeling, spatial regression did not capture significant interindividual variability. Specifically, predictions of the uncentered gradients had larger errors compared to the group average (Supplementary Figure1; Supplementary Table 2), indicating that individual geometry does not capture sufficient functional variability to explain differences in large-scale gradient distribution.

### Control analyses confirm dependence on individual cortical geometry

Control analyses demonstrated that the effects of the centered models depend on subject-specific geometry. To test this, we re-evaluated the same spatial models after replacing each subject’s embedding coordinates with the group average or randomly permuting embeddings across individuals. Then, we assessed the effect of combining these perturbed predictions with the individual gradient baseline as before. In both cases, the searchlight-level errors significantly increased compared to using true individual geometry (Fig. 2A). In fact, combining these control estimates with the individual gradient baselines increased their errors (Fig. 2A). Thus, the variability captured by the spatial models cannot be attributed to the general layout of vertices on the surface mesh, nor to a blurring effect. Instead, the observed error reductions depend on the true correspondence between individual intrinsic geometry and functional gradients.

### The contribution of geometry is strongest in regions of functional transition

Next, we ran vertex level tests to outline the regions where spatial model predictions most steeply reduced the errors of the individual gradient baselines (Fig. 2B). These tests were consistent with the whole-cortex findings, yielding widespread significant results. The principal gradient showed the most widespread significant results, covering 48% of vertices in the left hemisphere and 49% in the right. Gradient three followed with 42% and 45% respectively, while only 24% and 27% of tests were significant for gradient two.

**Figure 3 |:**
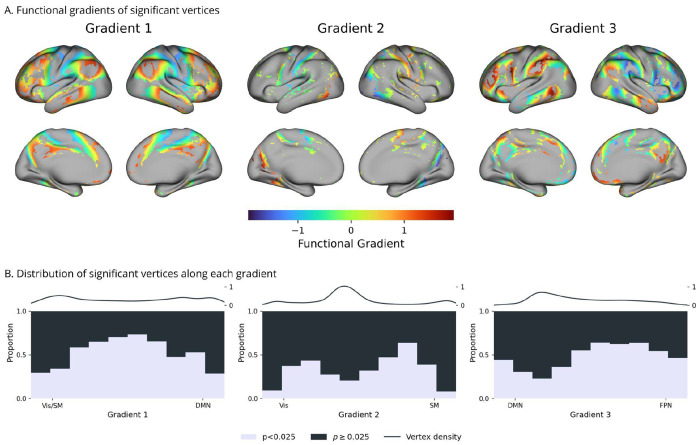
Distribution of significant results along the gradients **A.** Functional gradients masked to show only significant vertices. Error reductions associated with individual geometry encompass areas of gradient transition. **B.** Histograms of the fraction of significant vertices within successive gradient bins. Significant contributions of cortical geometry involve areas with neither a markedly unimodal, nor transmodal functional profile.

Significant results had distinct spatial distributions across gradients (Fig. 2B and Fig. 3A), but they overlapped over the parieto-occipital sulcus, the pre- and post-central sulci, and the posterior temporal cortex. For gradient one, significant vertices concentrated along the borders of the primary somatosensory and motor cortex, extending into posterior parietal and temporal regions, including also the posterior cingulate and precuneus. For gradient two, effects were more limited, involving only the borders of the visual cortex, and the outer borders of the precentral and postcentral gyrus. For gradient three, significant vertices were located in the middle temporal gyrus, superior parietal lobule, lateral and medial prefrontal cortex, and precuneus.

To better contextualize the vertex level results, we examined where significant geometry–function associations occur along the three functional gradients (Fig. 3B). For each one, we plotted the proportion of significant vertices within successive bins to understand how error reductions relate to position in the functional space defined by gradients. For gradient one, most significant results were found near the component’s mid-range corresponding to transitions between unimodal and transmodal regions. For gradient two, significant effects were located near intermediate positive and negative sections, along the borders of the visual and somatomotor cortex. For gradient three, significant results mainly involved its positive half, with a narrow spike near its negative end. In terms of canonical resting-state networks, these distributions cover areas in between sensory systems and the default-mode network, which mostly correspond to the attention, and the frontoparietal networks (Fig. 3B). However, the lateral parietal and temporal nodes of the default mode network also hosted some significant results.

Furthermore, we also replicated the control tests comparing the use of true individual geometry against group average and shuffled embeddings. These also confirmed previous results, with the true predictions performing better than the perturbed ones for the majority of the cortex. Furthermore, t-values from the permutation test were strongly correlated with those of the first vertex-wise comparisons (Fig. 2C, blue). Thus, the contribution of cortical geometry was strongest where predictions were most sensitive to the permutation of embeddings, further highlighting the association between true individual geometry and function.

On the whole, cortical geometry was most relevant near gradient transition zones, where vertices smoothly switch their functional affinity between different systems (Fig. 3A). At the gradient extremes, instead, associations between geometry and function were proportionately fewer, consistent with the low spatial variability of gradients in these regions. Together, these results show that individual differences in cortical geometry contribute to how functional transitions unfold on the cortex, shaping their spatial rate and direction across individuals.

### Effect of geometry at different distance ranges

**Figure 4 |:**
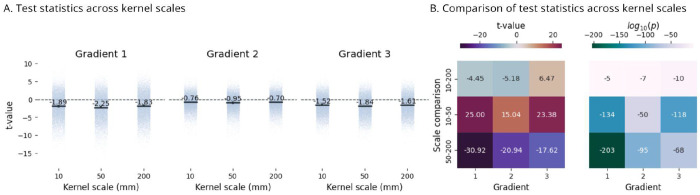
Error reduction across kernel scales **A.** Mean t-values (and 95% confidence intervals) for each gradient and kernel scale. **B.** Test statistics from the comparison of error reductions across kernels. Embeddings emphasizing mid-range geometry (scale = 50 mm) yield the best results.

Because intrinsic geometry can be defined at different spatial scales, we repeated the analyses using geometry embeddings that emphasized distances within a progressively larger radius: 10 mm, 50 mm, 200 mm. These embeddings capture short-, mid-, and long-range spatial relationships, allowing us to test how function relates to geometry at different scales. While the results presented so far are relative to the mid-range kernel, the overall patterns were consistent across scales. Regardless of scale, the predicted centered gradients significantly reduced the error of the individual gradient baselines when the two were combined. However, we found small but systematic differences in the strength of this effect, defined as the t-values of the error comparisons (Fig. 4A). Specifically, we found that the 50 mm kernel produced the greatest error reductions. Less pronounced differences were also observed between the 10 mm and 200 mm kernels. Short-range distances performed better than long-range distances for gradient one and two, while the opposite was found for gradient three (see Fig. 4B for all pairwise test statistics). Together, these results suggest that short- to mid-range spatial relationships are the most relevant to determine individual differences in functional organization.

## Discussion

Our results show how individual differences in cortical geometry are systematically associated with individual differences in local functional organization. We modeled the relationship between locally centered functional gradients and intrinsic cortical geometry. Through this, we demonstrate that geometric variability explains how functional transitions unfold across the cortical surface of different individuals. In other words, the rate and direction of transition between large-scale functional systems depend on individual cortical shape, while their overall spatial layout is largely conserved across individuals. Further, we demonstrate that this relationship hinges on individual-specific topography. Averaging or shuffling geometry embeddings across individuals breaks the correspondence between individual geometry and function, nullifying our results. Overall, short-to-mid spatial scales yielded the best results reflecting the established scales of spatial dependency in connectivity^15,20^, and bridging previous knowledge on within-surface functional autocorrelation with interindividual variability. Collectively, these findings suggest that cortical geometry contributes to individual functional differences by shaping functional transitions between functional systems.

Dense local connectivity underlies the association between cortical shape and function^21^. Local projections decay exponentially with distance^20^ imposing distance-dependence on the propagation of neural activity. This is reflected by the spatial autocorrelation of functional connectivity^15^, which links functional profiles to vertex positions relative to the rest of the cortex. Previous studies have demonstrated that distance-dependence explains functional differentiation within the individual cortex, and that it closely relates to the spatial distribution of the first functional gradient^5,6,15^. By fitting cross-subject spatial models we show that functional variability can be partly explained by the same spatial constraints. In line with this, we observe that relevant differences in intrinsic cortical geometry are best captured by distance within a 10 mm and 50 mm range, which matches the known range of autocorrelation in structural and functional connectivity ^15,20^. In other words, we show how within-cortex functional autocorrelation translates into interindividual variability in cortical organization.

Because single, cross-individual models partly explain gradient variation, we conclude that cortical geometry constrains local organization in a consistent, generalizable way across individuals. As a consequence, functional transitions behave as individualized realizations of the same spatial process. The spatial structure of this process captures the dependence of functional differentiation on spatial relationships within the intrinsic cortical space. Thus, our study supports the view of cortical geometry as a universal organizing principle and shows how it could determine interindividual differences in functional organization.

Consistent with previous studies^11,12^, we found that individual geometry does not outperform group average data in explaining differences of macroscale gradient distribution. Because the general shape of the cortex is highly conserved in the population, its large-scale effects may also be conserved between individuals. Hence, at this scale, the ability of geometry to explain individual functional differences may be limited. Such effects, instead, emerge when comparing drastically different cortical morphologies. Across species, for example, the association between global cortical organization and geometry becomes more readily observable as the shape and functional properties of the cortex adapt to different ecological niches^22,23^. Together, these observations would explain why we have found an association between geometric and functional variability at the local but not global level.

Our analyses suggest that geometry-related functional differences emerge at a finer spatial scale and are relational in nature. This is highlighted in our analyses by residualizing gradients against local individual means. This step also helped account for potential additional factors contributing to functional variability. Previous studies have suggested that geodesic distance interacts with other mechanisms to determine functional organization within individuals and at the group level. Thalamic connectivity, for example, interacts with molecular gradients—a space-dependent process—to initiate cortical arealization at the early stages of development^24^. Similarly, the interplay between geodesic distance and long-range structural connectivity has a key role in determining within-cortex functional variation^14^. In line with our results, such interactions appear to be most critical precisely in primary sensory and higher association areas^13^. Thus, while explicitly modeling additional features is beyond the aims of this paper, we propose that individual gradient baselines likely capture these and other factors. In this way, our study indirectly expands on such evidence, showing that, after accounting for locally uniform individual effects, the residual interindividual variance is associated with geometry.

Prior studies suggested that functional gradients emerge from geometric properties of the cortex, which guide the decay of influence of predetermined functional anchors^3,25^. consistent with this hypothesis, we show that cortical geometry is associated with the spatial rate of transitions at the interface of the unimodal and transmodal systems. Regions that occupy this section of the gradient space (parietal-temporal junction, superior parietal lobule, lateral prefrontal cortex) display well-documented functional heterogeneity^26–28^. Specifically, the frontoparietal and attention networks contain distinguishing features of the individual functional fingerprints ^29^. The intermediate sections of the gradients also show a lower heritability of functional connectivity, which could be contributing to the lack of significant effects over most of the purely sensory or association regions^30^. Together, this evidence suggests that spatial constraints effectively shape individual variability of functional topography away from strongly pre-determined regions of the cortex.

The core limitation of this study is that, despite demonstrating a geometry-function association, it does not provide a fully predictive model of functional gradients. Thus, our findings apply specifically to relative functional differences within the local functional context. The associative nature of the results also implies that they do not prove a causal mechanism.

Other limitations are related to the gradient centering. This methodological choice is similar to introducing subject level random intercepts to the model. Individual gradient baselines account for potential confounding variables but do not model them explicitly. Thus, this correction does not discriminate between purely space-independent information (e.g., age, sex) and phenomena with very low spatial frequency. Individual gradient baselines may combine a range of other explanatory factors including demographic and neurological variables.

This method also limits the scale of the detectable spatial processes. Where the scale is larger than the searchlight size, all gradient values are very close to the individual gradient baselines. Centering the data, then, leaves little residual variance for lattice Kriging to model, affecting the likelihood of detecting significant associations. As a result, our analyses highlight regions with either faster or very consistent gradient transitions.

Finally, using only the first three geometric components may focus on relatively stable geometric features^31^. Individual variability at this dimensionality may not be sufficiently pronounced, and, since local geometry seems the most relevant for functional organization, higher rank eigenmaps may be more closely associated with vertex function. However, beyond three dimensions, Euclidean distance becomes less meaningful^32^, which would make spatial modeling less reliable and interpretable in a higher-dimensional embedding.

These limitations outline the need for future research pointing out the questions left open by our study. First, establishing whether the associations we found truly reflect causal processes will require testing whether longitudinal changes in geometry can explain functional changes. This could be investigated in longitudinal developmental data to show that geometry contributes to the emergence of functional organization during childhood^33^. Second, future research should work on a comprehensive account of the interaction between cortical geometry and the other sources of variability that contribute to the local individual means. Existing literature already points to several candidates, such as cortico-cortical and cortico-subcortical structural connectivity. How their interplay with geometric constraints affects interindividual functional differences, however, remains to be assessed.

Finally, it will be important, going forward, to examine the implications of our results for task-related activity. Functional gradients constitute the latent functional space wherein task-evoked dynamics emerge^34^, and they are associated with individual-level task activity ^12^. To better understand the implication of geometry-function relationships, future research should assess whether this association is driven by the effect of geometry on gradients.

Overall, our findings support the longstanding hypothesis that the emergence of cortical organization is subject to geometric constraints that shape its topography. We show that, by shaping local functional differentiation, these constraints help explain how functional architecture varies across healthy individuals. This contributes to disentangling stable organizational principles from individual variation of functional architectures, building towards a more comprehensive account of brain function.

## Methods

Our goal was to determine whether individual differences in cortical geometry are linked to differences in local functional organization. First, we summarized cortical organization using the first three functional gradients — the dominant latent components of functional connectivity (Fig. 1A). Next, individual surfaces were embedded into a shared geometric space, which captures variability in vertex position across cortices (Fig. 1B). Lastly, we defined geometry-dependent functional variation by modeling locally-centered gradients based on vertex location in this geometry embedding (Fig. 1C).

### MRI data

We analyzed resting-state fMRI data from 960 healthy participants (age: 22–35 years) from the WU-Minn Human Connectome Project^35,36^, excluding participants with reported anatomical anomalies. The recruitment procedures and informed consent forms for participants were approved by the Washington University Institutional Review Board (IRB) as part of the HCP. This sample was split into a training set for model fitting (N=719, 392 female), and a test set for evaluation (N=241, 123 female). For each participant, we analyzed four resting-state fMRI runs (~15 min each; ~60 min total) and corresponding reconstructed cortical surfaces. To preserve intrinsic cortical geometry while maintaining vertex correspondence across individuals, we used MSMAll-registered native surfaces^37^. Functional data were processed with the HCP minimal preprocessing pipeline^35^, including distortion and motion correction, field map correction, and projection to the fsLR32k cortical mesh. Time series were also smoothed (6 mm FWHM) and downsampled to 10,242 vertices per hemisphere to reduce computational costs.

### Functional connectivity gradients

We characterized vertex functional profiles by mapping each vertex to a coordinate system defined by the first three gradients of functional connectivity (Fig. 1A)^3,18^. Gradients reduce vertex connectivity to a small set of dimensions that capture how functional profiles vary across the cortical sheet. The location of vertices within this functional space reflects the relative affinity to different functional systems such as sensory, attention, and association networks. For each participant, we computed vertex-wise Pearson correlations across concatenated resting-state runs to generate individual connectivity matrices, which were thresholded to the 5% strongest connections. Individual and group-average matrices were decomposed together using the joint embedding framework, thus aligning individual gradients to a common group reference while preserving subject-specific variation^19^. We focused on the first three gradients, which represent established axes of cortical organization, spanning unimodal to transmodal, visual to somatosensory, and task-positive to task-negative regions.

### Shared geometry embedding

To compare the cortical location of vertices across participants, we projected individual cortical surfaces into a shared geometry embedding (Fig. 1B). The diffeomorphic spectral matching framework^38^ was adapted to jointly embed individual and reference geodesic distance matrices into a common latent space.

We first computed subject level matrices of geodesic distance between vertices and converted them to affinity using a Cauchy kernel. Different kernel sizes (FWHM = 10/50/200 mm) were applied to emphasize short-, mid-, and long-range spatial relationships. Next, we constructed a block matrix ***W*** for each individual, containing the individual and reference matrices on the diagonal and their geometric mean on the off-diagonal. Laplacian eigenmaps of ***W*** were then computed by solving the generalized eigenvector problem Ly=λDy—where ***D*** is the weighted degree matrix of ***W*** and $L=D^{-\frac{1}{2}}(D-W)D^{-\frac{1}{2}}$ is the normalized Laplacian of ***W***. The first three non-trivial eigenvectors were retained.

This shared geometry embedding provides a compact coordinate system representing intrinsic cortical geometry: vertices with similar coordinates occupy equivalent positions across individual surfaces. Modeling functional gradients in this space, we tested whether individual variability in functional organization follows systematic differences in cortical geometry.

### Spatial models of local and global functional organization

We used spatial regression (lattice Kriging) to assess whether functional gradient variability is associated with vertex location within the shared geometry embedding. This method models spatial data as a weighted sum of radial basis functions distributed on a regular lattice^39^. This provides an efficient representation of linear and nonlinear spatial structure in unevenly sampled datasets while accounting for nonstationarity (see Supplementary Methods and^39^ for more detail).

The geometry embedding was divided into non-overlapping cubic searchlights with sides four times the mean distance between neighboring vertices (Fig. 1C). The searchlights contained all vertices which fell within its borders across all participants, grouping together vertices that occupy similar cortical locations, irrespective of vertex identity. Defining the searchlights directly in the embedding space ensured consistency of the spatial domain in which spatial models were fitted.

Within each searchlight, functional gradient values were first centered at the subject level by subtracting the local individual mean (Fig. 1C). This helped control for individual gradient offsets independent from geometry, and isolated fine-scale functional deviations within the global gradient distribution. The resulting centered gradients retain the sign and magnitude of gradient differences on the cortical surface, reflecting rates and directions of local functional transitions on the cortex.

Using exclusively data from the train-set participants, a spatial model was fitted per searchlight using vertex coordinates in the geometry embedding as spatial predictors (Fig. 1C). This procedure was replicated for both centered and uncentered gradients, investigating geometry’s association with vertex location within local functional transitions and global functional architecture.

### Statistics

After fitting the spatial models on the subjects included in the training set, we applied them to the held-out subjects, the test set. All the following analyses, then, were performed on the resulting predictions.

#### Associations between gradients and cortical geometry

For centered gradients, we were interested in whether the spatial models capture meaningful additional variability beyond what the individual gradient baselines already account for. Thus, we compared the error of the baselines before and after adding Kriging predictions to them, for both the whole cortex and individual vertices. At the whole cortex level, we compared the grand mean of individual absolute errors across searchlights using t-tests for paired samples. At the vertex level, instead, we compared errors for a specific vertex using one-tailed t-tests for paired samples across individuals. At this level, to prevent results from being driven by vertices whose spatial prediction was based on more datapoints, individual samples were weighted by the variance of their vertices’ gradients in the searchlight.

For the uncentered gradients, we performed analogous comparisons, but tested whether geometry-based predictions were more accurate than the group-average gradients. Both analyses were conducted at whole cortex and vertex levels as above.

All p-values were corrected for multiple comparisons using FDR—across comparisons for whole cortex tests, and across comparisons and vertices for vertex level tests.

#### Dependence on individual geometry

To test if the spatial models captured variance specific to individual cortical geometry, we re-evaluated the centered gradient estimates after replacing each subject’s geometry embedding with the group mean while keeping model parameters fixed. Higher prediction errors after this substitution indicated that average cortical geometry could not account for gradient deviations from local subject means.

As an additional control, we also repeated the evaluation after permuting vertex embedding across subjects 100 times. Comparing the mean error of these predictions with the true model ensured that results from the previous test were not determined by average geometry being too different from true cortical surfaces.

Both tests were performed at the whole cortex and vertex level as in the main analyses. At the whole cortex level, we also compared the error of the subject means before and after adding the group-mean-based and permutation-based predictions. This was done to test if any form of spatial interpolation could determine an improvement compared to the subject means.

#### Contribution of different distance ranges

Finally, we assessed the replicability of the analyses across the spatial scales used for the geometry embedding. All analyses were repeated with embeddings computed using the 10, 50, and 200 mm kernels, representing short-, mid-, and long-range geometric relationships. We then compared the distributions of the main vertex-wise test statistics—improvements over subject means and group-average gradients—across kernel sizes to assess how embedding scale affected model accuracy.

## Supplementary Material

This is a list of supplementary files associated with this preprint. Click to download.

- SupplementaryInformation.docx

## Data Availability

Data were provided by the Human Connectome Project, WU-Minn Consortium (Principal Investigators: David Van Essen and Kamil Ugurbil; 1U54MH091657) funded by the 16 NIH Institutes and Centers that support the NIH Blueprint for Neuroscience Research; and by the McDonnell Center for Systems Neuroscience at Washington University. All data are obtainable from the HCP website (https://db.humanconnectome.org/).

## Code Availability

The HCP preprocessing pipelines can be found at https://github.com/Washington-University/HCPpipelines, while the code used to run all subsequent analyses is made available as an installable package on F.A.’s GitHub repository https://github.com/alberti-f/VarioGrad.
